# Alzheimer’s disease and related dementia: evaluation, diagnosis and acute care management

**DOI:** 10.3389/fneur.2026.1743770

**Published:** 2026-06-15

**Authors:** Rade B. Vukmir

**Affiliations:** Department of Emergency Medicine, Drexel University, Philadelphia, PA, United States

**Keywords:** Alzheimer disease, biomarkers, delerium, dementia, imaging, cognitive impairment

## Abstract

**Importance:**

The patient presenting with memory loss often requires a complex, extensive multidisciplinary specialty evaluation that may begin in the primary care, emergency department, or general neurology setting. The analysis begins with a suspicion or concern regarding cognitive performance raised by the patient, family, or provider. Ideally, a better understanding will empower the primary care and general neurology communities to screen for and appropriately diagnose, treat, or refer patients with dementia.

**Methods:**

The thematic focus of this narrative review is diagnosis, imaging, and treatment of Alzheimer’s disease and related dementia (ADRD). Information was abstracted from the National Library of Medicine MEDLINE/PubMed database. Medical Subject Headings (MeSH) heading search terms included dementia and, more specifically, Alzheimer’s disease. The search targeted primary research, preferentially compared to reviews, consensus statements, or case reports if feasible.

**Observations:**

Delirium typically represents an acute or subacute fluctuating change in mental status, often temporally related to acute illness. While dementia is typically associated with a more chronic progressive presentation of cognitive change without the presence of concurrent illness. However, subacute or dementia presentations may be exacerbated in that setting as well.

**Conclusion and relevance:**

The diagnosis, management, and therapy of Alzheimer’s disease and related dementia is undergoing rapid change in imaging and now the utility of blood-based biomarkers. As more amyloid-modifying therapy is administered, the acute care systems should be knowledgeable of the treatment course and potential for complications.

## Background

First, there is an age-related effect on cognition or memory loss that may have a wide variability in progression among patients (1). In fact, in normal individuals, there is an inflection point at just before 50 years of age, where “crystallized” cognitive abilities, such as vocabulary, continue to improve slightly or stabilize before decline, while “fluid” cognitive abilities, such as processing speed, begin a slow but steady decline over time (2). Therefore, even with individual variation, age-related decline may begin earlier in life than expected.

Second, the patient may express concerns about changes in their cognitive performance, only perceptible to them, known as subjective cognitive impairment (SCI). Third, the patient, family, friends, or coworkers may notice an objective change in memory, capability to perform, known as objective cognitive impairment (CI)-classified as mild (MCI) to moderate. Finally, the patient may manifest severe cognitive changes that significantly impact function consistent with the diagnosis of dementia.

### Definition

Dementia manifests as a constellation of symptoms related to memory, executive function, and functional capability that is severe enough to affect the activities of normal daily functioning. Memory loss typically occurs in a retrograde fashion, characteristically progressing from recent to past memory. While Alzheimer’s disease is manifested as the symptoms of dementia that are accompanied by neuronal degeneration with characteristic insoluble beta amyloid (AB) deposits combined with phosphorylated tau protein (p-tau) and neurofibrillary tangles (NFTs), it ultimately destroys the normally functioning neurons.

### Incidence

Dementia onset has a clear age progression, with estimates of incidence doubling every 5 years in later life. There are estimates of a one-third prevalence in those over 85 years of age. Age-specific stratification defines a dementia incidence of 1.7% for those 65–74 years, 5.7% for those 75–84, 13.1% for those greater than 85, and 33% for those greater than 90 years of age (3).

Higher prevalence was again noted in those patients who were older, had less educational attainment, had an annual income <200% federal poverty level, were racial and ethnic minority groups, and those who are widowed, divorced, or never married. Individual variation is acknowledged, with some individuals demonstrating lesser or greater cognitive resilience (4).

### Etiology

Alzheimer’s disease is the most common etiology of dementia, both by incidence and in the public perception. However, other defined causes include vascular dementia associated with microvascular disease or large vessel occlusion. Vascular dementia is typically more acute in onset or temporally related to a cerebrovascular event or events. Lewy body dementia (LBD) is caused by alpha-synuclein deposits, a variant associated with Parkinson’s disease. There are characteristic movement disorders, difficulty with balance, sleep disturbance, and vivid visual hallucinations. Frontotemporal dementia (FTD) is defined by a characteristic presentation in which early, subtle personality and behavioral changes before more objective indices of loss of function.

### Risk factors

Dementia is felt to correlate with the normal aging process in some, with a progressive increase in incidence involving approximately one-third of those patients over 85 years (5). The incidence of Alzheimer’s dementia (AD) at age 65 years is less than 5%, increasing to more than 40% in those patients beyond age 85 years (6).

The APOE4 allele in both heterozygotes and homozygous conditions confers a familial predisposition with early onset disease, particularly in individuals under 60 years of age. In addition, the overall incidence of complications, such as ARIA-E, was higher in APOE-e4 carriers than non-carriers and occurred more often in homozygotes than heterozygotes (7).

There is a strong demographic influence where African American patients are twice as likely, and Hispanic patients 1.5 times as likely as their white counterparts to develop Alzheimer’s dementia (8). This finding should stress the importance of focusing on care equity.

There are over 400 other potential Alzheimer’s disease correlates, including hypertension, diabetes, obesity, head injury, social isolation, and depression among others (9).

### Presentation

Patients are often encountered in the primary care or ED setting at different stages of dementia development. Here, early presenters may present as high functioning at baseline with subtle concerns over higher executive function deficiencies, including personality changes, complex problem-solving, and decision-making difficulty first noticed by coworkers. While later presenters may present with memory loss, driving difficulty, vocational or hobby task completion, or inability to perform activities of daily living, as reported by family.

The patient’s family typically presents with concerns over agitation, functional or behavioral changes, often exacerbated by the loss or debilitation of the patient’s partner. Oftentimes, slow decline is compensated for and masked by the patient’s significant other.

The key to any subsequent dementia workup, including potentially biomarker testing, imaging, or referral, is to define a high pretest-probability cohort to ensure reliable testing and advance diagnostic accuracy.

### Genetic testing

The focus of genetic testing is apolipoprotein-E (APOE-4), a gene coding for a cholesterol-carrying protein (10). Analysis of APOE variants finds the APOE-e2 allele occurring in 5–10% of patients may have a protective function or be associated with a delayed onset of dementia. While the APOE-e3 appears to have no actual effect on subsequent disease incidence.

Finally, APOE-e4 is associated with a higher risk of earlier onset dementia, especially with the homozygous condition (10). The heterozygous allele is found in 15–25% of individuals, and homozygous alleles in 2–5% of individuals. The majority, 90% of patients, are afflicted with normal onset (>65 years) Alzheimer’s; while the minority, 10%, are afflicted with early onset (<65 years) Alzheimer’s dementia.

In addition, there are single gene variants associated with abnormal amyloid deposits, breakdown, and plaque formation. These direct genetic mutations include amyloid precursor protein (APP) located on chromosome 21, presenilin 1 (PSEN1) on chromosome 14, and presenilin 2 (PSEN2) on chromosome 1.

Finally, patients affected by Down’s syndrome, where there is an extra copy of chromosome 21, featuring APP, establish additional dementia risk. Here, there is a 50–60% increase in lifetime dementia risk, typically with early onset occurring between 50 and 60 years of age.

Here, genetic sampling should be performed in those with a clinical presentation consistent with early-onset dementia.

## Evaluation

### Laboratory

The change in mental status evaluation includes the standard laboratory testing, complete blood count (CBC), comprehensive metabolic profile (CMP), liver function tests (LFTs), and coagulation indices.

Targeted screening for infectious conditions, may include rapid plasmin regain (RPR) and human immunodeficiency virus (HIV); endocrine, may include thyroid function testing (TFT), cortisol, and prolactin; hepatic conditions, may include ammonia, ceruloplasmin in Wilson’s Disease, iron, ferritin, and transferrin in hemochromatosis; inflammatory conditions, may include erythrocyte sedimentation rate (ESR) and c-reactive protein (CRP); toxicology, drug screen and heavy metal screen.

Lumbar puncture and cerebrospinal fluid (CSF) sampling are the gold standard for Alzheimer’s diagnosis, assessing the presence of neurological injury and markers of infection, inflammation, and malignancy.

### Imaging

**Figure 1:**
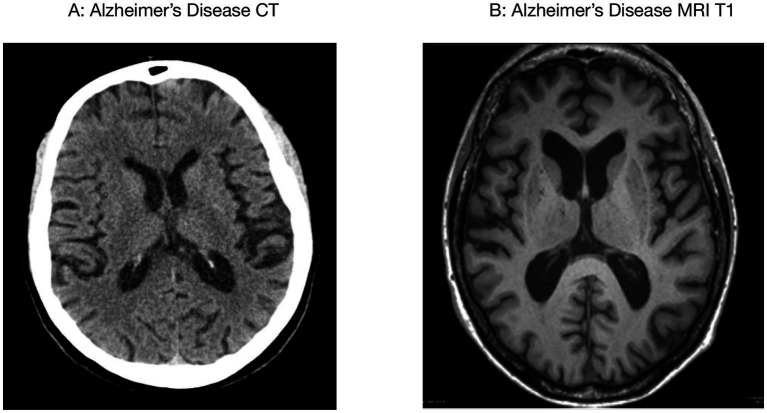
**(A)** Alzheimer’s disease CT. **(B)** Alzheimer’s disease MRI T1.

The earliest neurological imaging includes computed tomography (CT) to rule out hemorrhage, with contrast added to evaluate for infection, inflammation, or malignancy. However, CT findings can include enlargement of cerebral sulci, loss of gyral volume, followed by progressive atrophy in the medial temporal, parietal, and eventually frontal regions. This progressive change may then be accompanied by ex-vacuo compensatory ventricular dilatation and hydrocephalus (Figure 1A) (11).

**Figure 2:**
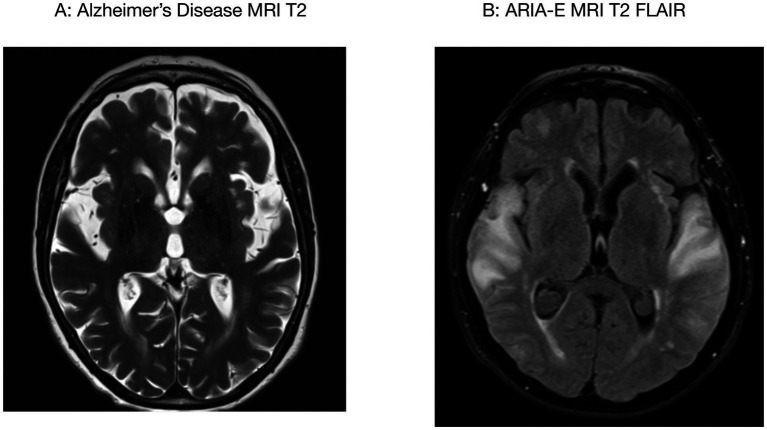
**(A)** Alzheimer’s disease MRI T2. **(B)** ARIA-E MRI T2 FLAIR.

The next stage of imaging should include magnetic resonance imaging (MRI), initially used for screening to exclude inflammation, infection, cerebrovascular events, or malignancy, which is better visualized with T2 sequencing that emphasize the water signal. While the selective atrophy of particular brain regions associated with dementia can be better delineated with T1 sequencing emphasizing fat signal (Figures 1B, 2A) (12). Initially, in dementia, the sulci and lateral ventricle temporal horns appear to be more prominent, with subsequent reduction noted in hippocampal and amygdala regions.

**Figure 3:**
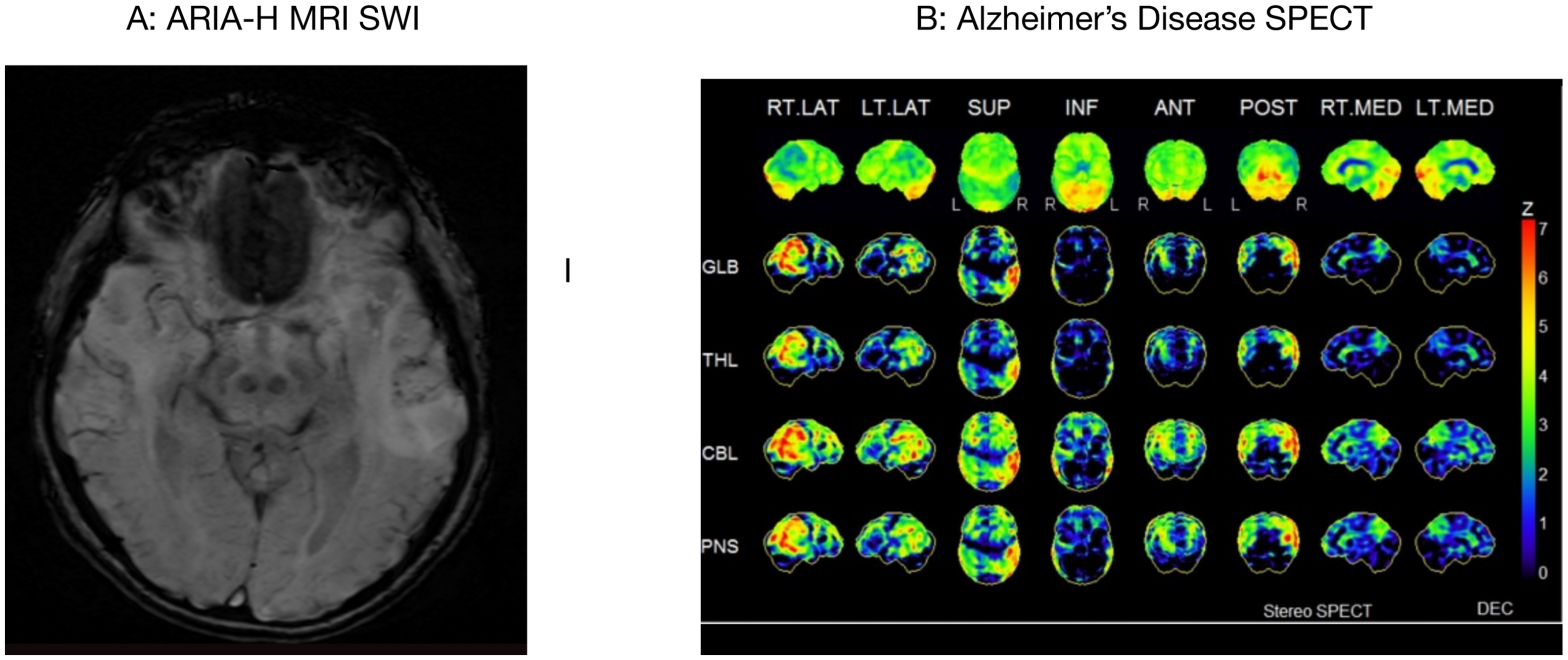
**(A)** ARIA-H MRI SWI. **(B)** Alzheimer’s disease SPECT.

However, it does utilize T2/fluid-attenuated inversion recovery (FLAIR) imaging sequences to define amyloid-related imaging abnormalities (ARIA-E) and T2/(gradient echo) GRE and susceptibility weighted imaging (SWI) defining ARIA-H (Figures 2B, 3A) (13). ARIA-E manifests as cortical and subcortical vasogenic edema localized initially to the temporal regions, as well as sulcal hyperintensities denoting effusions on FLAIR imaging. Similarly, ARIA-H noted as subcortical micro-hemorrhages, focused on the temporal region in SWI imaging. These lesions can occur in the setting of normal dementia disease progression or in the setting of amyloid-modifying therapy for Alzheimer’s disease.

A more sophisticated exam single photon emission computed tomography (brain-SPECT), a type of nuclear medicine study, combines both the structural and functional aspects of imaging, emphasizing the latter. Here, the subtle behavioral aspects of brain function are defined by correlating them with brain regions that are functioning properly, underactive, or overactive to assist in the diagnosis of some neurological and psychiatric conditions (Figure 3B) (14).

**Figure 4:**
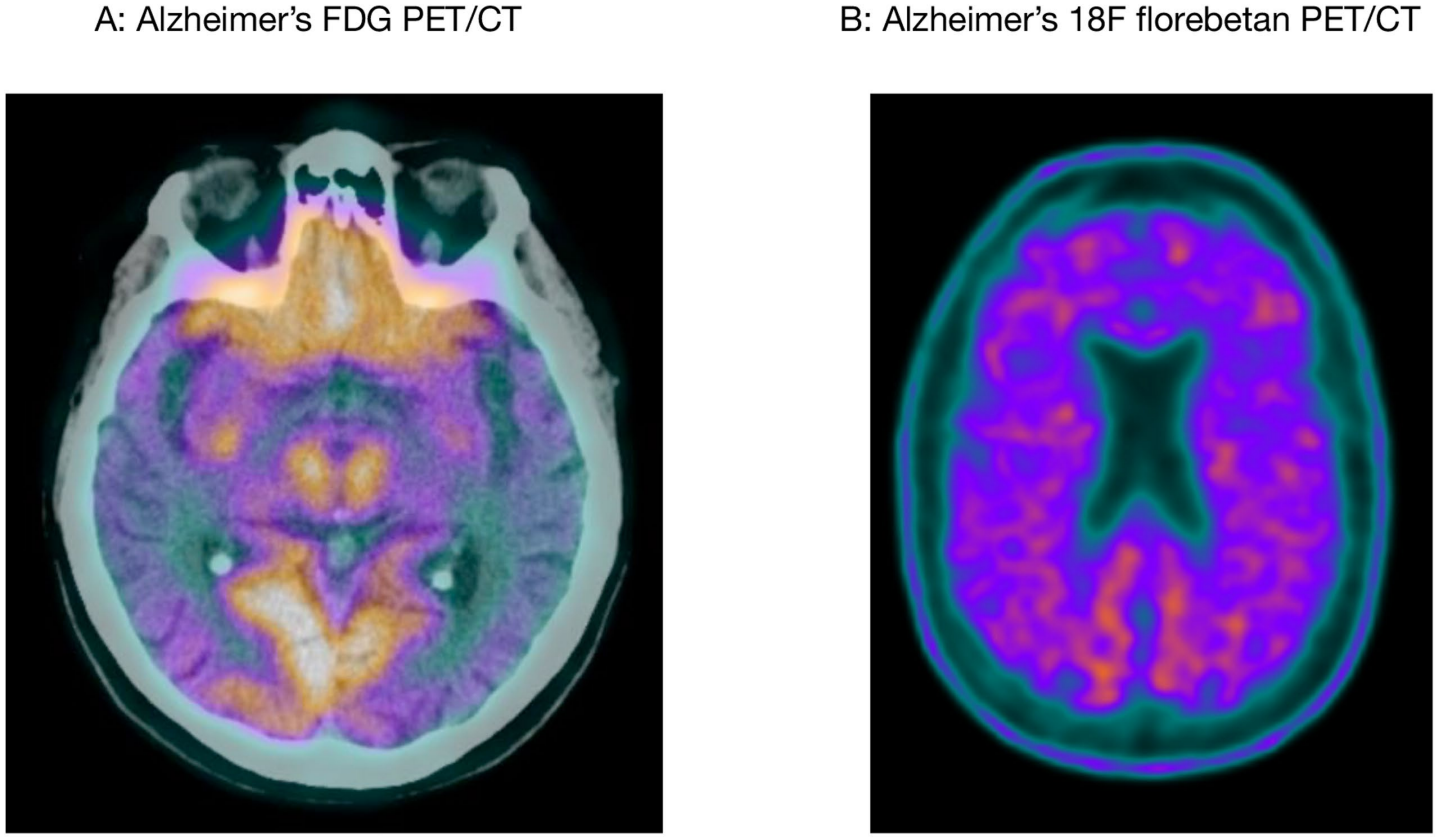
**(A)** Alzheimer’s FDG PET/CT. **(B)** Alzheimer’s ^18^F florebetan PET/CT.

However, the definitive diagnostic imaging utilized for the diagnosis of Alzheimer’s disease is positron emission tomography (PET). The most basic imaging technique (FDG-PET) utilizes a bound glucose tracer to define standardized uptake activity (SUV) and track the brain’s physiological activity, focused on areas of “cold” regions of decreased metabolic activity, specifically in the parietotemporal region. In addition, this imaging may be particularly helpful in diagnosing frontotemporal dementia (Figure 4A) (11). As well, amyloid PET or Tau PET, such as ^18^F Florbetaben PET/CT imaging, focused on “hot” regions can illustrate areas of pathological uptake, indicating amyloid deposits or tau protein tangles respectively, consistent with the diagnosis of Alzheimer’s dementia (Figure 4B) (15).

### Cerebrospinal fluid analysis

Until now, the definitive Alzheimer diagnosis has been facilitated by identifying specific biomarkers, beta amyloid 42 and AB1-42/AB1-40 ratio decline, indicating the presence of beta amyloid plaque. The AB 1–42 fraction accumulates in senile plaques, while the 1–40 fraction is unchanged, the ratio decrease is felt to be more specific (16).

Similarly, the presence of total tau initially and later phosphorylated tau (181)isolated from tau tangles correlated with abnormal amyloid on PET imaging, and was felt to be indicative of the presence of AD (17). Subsequently, plasma p-tau 217 was felt to be comparable to CSF biomarkers to determine abnormal amyloid and tau pathology consistent with AD (18).

### Cognitive screening evaluations

The emergency medicine practitioner should be familiar with commonly used comprehensive cognitive screening tools such as the Mini-Mental-State Evaluation (MMSE) and the Montreal Cognitive Assessment (MoCA) screening (19, 20). However, it is recognized that these comprehensive screening tools are not appropriate for acute or primary care settings and are best performed by experienced neurocognitive specialists to provide the most accurate assessment. Here, they would evaluate more nuanced cognitive areas, including memory, language, attention, judgment, and executive function.

The MMSE, developed by Folstein et al. (19), defines scores in the 25–30 range as normal, inversely proportional to patient age, with scores of 24 or less considered abnormal. They examine five domains of cognition, including orientation, memory, attention and calculation, language, and design copying.

The MoCA developed in 2005 has a normal range of 26–30, with scores of 18–25 indicating mild, 10–17 moderate, and <10 severe cognitive impairment (20). They examine eight knowledge and skill domains to include visuospatial/executive, naming, memory, attention, language, abstraction, delayed recall, and orientation (21). This exam is felt to be more sensitive, denoting smaller degrees of impairment in MCI, 90 vs. 18% compared to MMSE.

However, acute care providers are capable of performing a validated 3-min brief cognitive screening, typically the MiniCog (0–5), including three tasks: word registration, clock drawing, and word recall, with abnormality indicated by a score <3 (22). Similarly, the Mini-MoCA utilizes the same 30-point scoring system, but suggests a shortened performance time from 10 to 5 min required (23).

Meanwhile, the primary care community often utilizes the General Practitioner Assessment of Cognition (GPCOG) (scoring 0–9). The GPCOG utilizes an initial cognitive assessment, including name and address recall, time-date, clock, and current news information, followed by a family informant six-item questionnaire, if patient screening is equivocal or positive (24).

In a primary care setting, the testing should include a rapid screening test, preferably the MiniCog (3 min) or MiniMoCA (5 min), followed by MoCA if screening is abnormal at the subsequent visit.

### Blood-based biomarkers (BBMs)

The availability of plasma-based biomarkers commercially available in 2025, such as AB-42/40 and phosphorylated tau (p-tau), glial fibrillary acidic protein (GFAP), and neurofilament light chain (NfL), will help make the AD diagnosis more patient accessible in austere care environments (25) (Table 1).

**Table 1:** Alzheimer’s disease targeted blood based biomarkers.

Biomarker	Pathological target
Amyloid accumulation	
Amyloid-B	Amyloid burden
AB 1–42/1–40	
Phosphorylated tau	Amyloid plaque
p-tau 217	
p-tau 181	
p-tau 231	
Axonal neurodegeneration	
Neurofilament light chain	Neuroaxonal degeneration
NfL	Disease modifying therapy monitoring
Astrocyte	
Glial fibrillary acidic protein	Astrocyte activation
GFAP	Early change

References: (25, 26).

The MEMENTO cohort of 2,277 patients demonstrated that all blood biomarkers, except for total tau, were mildly correlated with CSF levels (*r* = 0.33 to 0.46, *p* < 0.0001) and were associated with amyloid PET status (*p* < 0.0001) (26). They identified p-tau 181 as the biomarker most closely correlated with amyloid PET positivity (AUC = 0.74 95% CI = 0.69; 0.79).

Subsequently, plasma p-tau 217 was considered more comparable to CSF biomarkers for determining abnormal amyloid and tau pathology consistent with AD diagnosis, as defined in longitudinal cohorts (18). High accuracy was noted identifying AUC (area under the curve) for elevated amyloid AB (AUC, 0.92–0.96; 95% CI, 0.89–0.99) and tau pathology (AUC, 0.93–0.97; 95% CI 0.84–0.99) in this cohort.

As well, blood-based biomarkers can be utilized for early disease monitoring. A cohort study of 298 memory clinic patients with subjective cognitive decline (SCD) measured amyloid B 42/40, p-tau 217, NfL, and GFAP (27). The amyloid positive group had higher baseline and steeper slope increases over time of p-tau 217, NfL, and GFAP levels. While the longitudinal increases in p-tau 217 and GFAP were associated with cognitive decline across all measured domains.

Remember, these blood-based biomarkers are not meant to be generalized “screening” tests and should only be ordered where clinical suspicion and pre-test probability for Alzheimer’s dementia is high. However, they may be useful for testing in more austere care environments with less access to PET imaging and specialty dementia care referral.

### Diagnostic pathway

The proper diagnostic approach is to screen high-risk individuals based on clinical concerns. Ideally, patient history augmented by family comments regarding cognitive change specifics and the rapidity of decline.

At-risk patients should have alternative illnesses excluded first. This should be followed by rapid cognitive screening in a primary care setting, initially, the Mini-Cog or Mini-MoCA evaluations.

This should be followed by a conventional cognitive screening exam, such as the MoCA evaluation, to detect mild cognitive impairment or dementia. This should ideally be performed at a subsequent visit, and potentially by a professional trained in cognitive exam evaluation.

Imaging should initially focus on condition exclusion, with a CT scan focused on hemorrhage and an MRI targeting ischemia, infection, malignancy, or cellular abnormalities.

The advent of blood-based biomarkers may allow screening the moderate to high prevalence population after the initial imaging step. Although commercially available blood sampling identifying proteins, such as amyloid B 42/40, p-tau 181, p-tau 217, GFAP, or NfL, are not all covered by Medicare or private insurance, they may be more accessible than some more sophisticated radiologic imaging (28).

This approach primarily targets those with clinical suspicion and those beyond middle age (50–55+) for testing. This involves single marker-p-tau 217 (ALZpath Dx), p-tau 181 (Roche); ratios-p-tau 217/AB 1–42 ratio (Lumipulse G), amyloid probability score AB ratio 42/40/%p-tau 217 (Precivity AD2), and combination marker panel testing—p-tau 217, AB 42/40, NfL, GFAP (LucentAD Complete) (Table 2) (28–32).

**Table 2:** Commercially available Alzheimer’s disease targeted blood based biomarkers.

Product	Target	Manufacturer	Sensitivity (%)	Specificity (%)	NPV (%)
Lumipulse G	p-tau 217/AB1-42	Fujirebio	97.6	90.8	97
ALZpath Dx	p-tau 217	ALZpath	95	88	95
Elecys	p-tau 181	Roche	91	69.8	96
Precivity AD2	Amyloid Probability	C2N	90–92	92	91
	Score (APS)				
	AB ratio 42/40				
	%p-tau 217				
LucentAD Complete	Amyloid risk score lucent	NfL	90	90	87
	p-tau 217				
	AB 42/40				
	GFAP				

References: (28–32).

There is slight product performance variability, but a focus on the test’s negative predictive value greater than 90% is likely to rule out the presence of abnormal amyloid plaque on amyloid or tau PET CT scan.

The protocol would assess pretest probability assessment, negative testing warrants continued observation, intermediate testing results would warrant interval repeat testing, and positive testing results would typically warrant specialty referral.

The most widely accepted diagnostic standard is to utilize amyloid or tau PET imaging or CSF biomarker sampling. Disadvantages include the fact that the former is a more costly, less available procedure, while the latter requires an invasive procedure.

Therefore, the utility of BBMs is accessibility to more patients, availability to general practice providers, and cost advantages.

### Equitable care access

There is a clear association between lower socioeconomic status (SES) and adverse outcomes in dementia. Multidimensional poverty is associated with a significant increase in dementia incidence, 2.3-fold in low- to middle-income countries (33).

As well, among Medicare beneficiaries there is a difference in the timely diagnosis of MCI or dementia incidence in white, compared to Asian (OR, 0.46; 95% CI, 0.38–0.56), Hispanic (OR, 0.62; 95% CI, 0.52–0.72) and Black (OR, 0.73; 95% CI, 0.56–0.94) (34). This difference was associated with fewer diagnostic elements in Asian beneficiaries (IRR, 0.81; 95% CI 0.74–0.87).

There is a clear imperative to ensure equitable access to dementia care across all socioeconomic, racial, and ethnic groups. Issues include primary care provider access, specialist availability, laboratory services, and sophisticated radiology imaging procedures.

Here, the importance of blood-based biomarkers is paramount, stressing the ease of access in the primary care setting, geographical availability in medical care deserts, and favorable cost comparison to imaging procedures.

### Treatment complications

Another critical area of knowledge development is the interface of Alzheimer’s disease pathology itself, or the use of disease-modifying treatment, and the interaction with unrelated therapeutic anticoagulation or anti-platelet interventions

### Amyloid-modifying therapy

The wider use of aducanumab before discontinuance, and now lecanemab and donanemab, warrants understanding of treatment and potential complications of the use of amyloid-modifying therapy (35–37). Amyloid-related imaging abnormalities (ARIAs), either vasogenic edema (ARIA-E) or hemorrhage (ARIA-H), may manifest in normal Alzheimer’s disease progression or disease treatment with amyloid-modifying therapy (38).

### Anticoagulation

The indication for anticoagulation for extraneous indications is critically important to consider in those cases where anti-amyloid therapy and the potential for ICH. A longitudinal cohort of 12,373 elderly, with an average age of 73 years, with 59% female, with MCI (20%) and dementia (9%) patients reported a 1-year risk incidence of new indication for anticoagulation (39). The indication for atrial fibrillation was 1.7, 1.7%; DVT 1.2, 1.8%; PE 04, 0.3%; AMI 1.2, 1.0%; stroke 2.0, 2.4%; and 5.7, 6.7% for any indication.

### Direct oral anticoagulant (DOAC)

Focus on the potential for interactions between antiplatelet agents, such as clopidogrel, anticoagulation, either conventional warfarin or direct oral anticoagulant (DOAC)–direct thrombin inhibitors (DTIs), such as dabigatran, or factor Xa inhibitors, such as apixaban or rivaroxaban.

A case was described where cerebral amyloid angiopathy (CAA) patient with atrial fibrillation and decreased mental status had an MRI T2 diagnosed with cortical microbleeds, and was prescribed a DOAC. Neurologic condition worsened with intracerebral hemorrhage after introduction and improved after DOAC was discontinued (40).

### Thrombolytics

However, the clinical scenario of most concern is the concurrent administration of intravenous thrombolytic medication in the stroke setting (41).

Typically, the use of single or dual antiplatelet agents or warfarin with PT < 1.7 is not an absolute contraindication to the administration of alteplase for cerebrovascular occlusion.

However, caution is warranted with the use of DOACs and intravenous TPA unless applicable hemostatic monitoring tests are normal or 48 h have elapsed since the last dose (42).

Similarly, the administration of intravenous alteplase in the setting of amyloid-modifying therapy or cerebral amyloid angiopathy (CAA) warrants extreme caution, in light of case reports of associated life-threatening intracranial hemorrhages (41). Specialty consult is warranted in the setting of an acute cerebrovascular event to decide on lower-risk treatment alternatives, such as intracranial thrombolysis or mechanical clot retrieval (43).

## Summary

Patients may often present initially with concerns over a change in cognition or dementia in the emergency or primary care setting. The acute care physician is adept at the altered mental status evaluation, including routine laboratory screening and head CT.

Referral to primary care or neurology providers typically includes a contrast-enhanced MRI to evaluate for inflammatory or infectious conditions. Finally, cognitive care specialists focus on standardized mental status testing, CSF biomarkers, and amyloid or tau PET imaging for Alzheimer’s disease diagnosis.

However, the advent of blood-based biomarkers to assist in the dementia diagnosis process is paramount to the practice of acute and primary care practitioners.

In the future, with proper clinical suspicion, blood-based biomarker testing may be utilized in conjunction with the radiographic imaging and the specialty referral continuum. A clinical scenario might include negative testing to continue to follow, an indeterminate result warranting a defined reevaluation interval (3–6 months), and a positive result referred for imaging and specialty referral.

Most importantly, in the setting of an acute cerebrovascular event, maximum care should be directed to minimize hemorrhage risk in those on anticoagulation, or scheduled to receive acute stroke intervention, such as systemic thrombolytic agents with advanced Alzheimer’s disease or in the setting of amyloid-modifying therapy.

## Conclusion

The increasing incidence of dementia creates ever-increasing burdens on patients, families, and the healthcare system. The healthcare system focus should include emphasis on primary care screening, blood-based biomarker access, understanding of focused radiological imaging, and better referral access to dementia specialty care.
